# Asymmetry of flow in aortic root and its application in hypertrophic obstructive cardiomyopathy

**DOI:** 10.1152/japplphysiol.00188.2023

**Published:** 2023-08-24

**Authors:** Nadine Francis, Mohammed Hosny, Magdi H. Yacoub, Kim H. Parker

**Affiliations:** ^1^Biomedical Engineering and Innovation Laboratory, Aswan Heart Centre, Department of Research, Magdi Yacoub Heart Foundation, Aswan, Egypt; ^2^Department of Bioengineering, Imperial College, London, United Kingdom; ^3^Department of Cardiology, Aswan Heart Centre, Magdi Yacoub Heart Foundation, Aswan, Egypt; ^4^Department of Cardiology, Cairo University, Cairo, Egypt; ^5^Department of Cardiac Surgery, Aswan Heart Centre, Magdi Yacoub Heart Foundation, Aswan, Egypt; ^6^The Magdi Yacoub Institute, Harefield Hospital, Harefield, United Kingdom; ^7^National Heart and Lung Institute, Imperial College, London, United Kingdom

**Keywords:** aortic root, flow asymmetry, healthy volunteers, hypertrophic obstructive cardiomyopathy, image processing

## Abstract

The aortic root (AR) performs sophisticated functions regulating the blood dynamics during the cardiac cycle. Such complex function depends on the nature of flow in the AR. Here, we investigate the potential of new quantitative parameters of flow asymmetry that could have clinical implications. We developed a MATLAB program to study the AR hemodynamics in each sinus of Valsalva using two-dimensional (2-D) cardiac magnetic resonance imaging during systole and particularly at peak systolic flow in 13 healthy volunteers and compared with 10 patients with hypertrophic obstructive cardiomyopathy (HOCM). We show that the effective area of the aortic jet in healthy volunteers is significantly higher at peak systolic flow and on average during systole. The flow asymmetry index, indicating how the jet is skewed away from the left coronary sinus (LCS), is small in healthy volunteers and much larger in HOCM at peak systole. The average of this index over systole is significantly more different between cohorts. Looking in more detail at the flow in the sinuses during systole, we show that the AR jet in healthy volunteers is more symmetrical, affecting the three sinuses almost equally, unlike the asymmetric AR jet in patients with HOCM that has decreased flow rate in the LCS and increased fractional area of backward flow in the LCS. The percentage of backward flow in the sinuses of Valsalva calculated over systole is a potential indicator of perturbed AR hemodynamics and the distribution of vortical flow and could be used as a measure of flow asymmetry.

**NEW & NOTEWORTHY** The aortic root is a vital organ responsible for performing sophisticated functions to regulate the blood flow dynamics during the cardiac cycle. Such synchronized complex performance affects and is affected by the flow symmetry and type of flow reaching the aorta. Here, we report flow asymmetry in the aortic root which could have clinical implications, and we investigate the potential of various quantitative parameters as measures of flow asymmetry in hypertrophic obstructive cardiomyopathy.

## Graphical abstract

**Figure:**
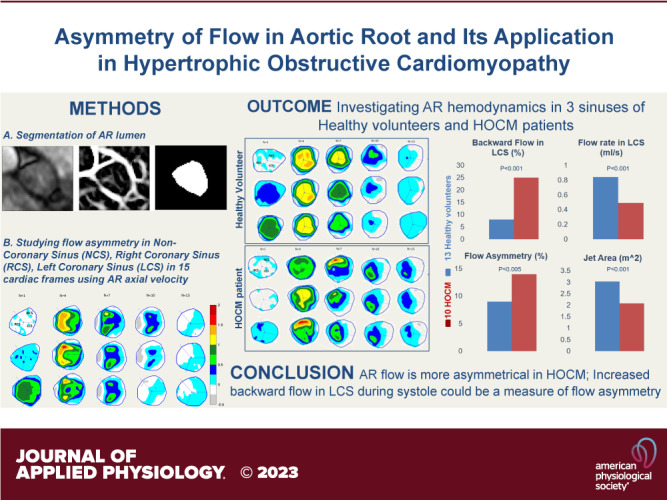

## INTRODUCTION

**Figure 1.**
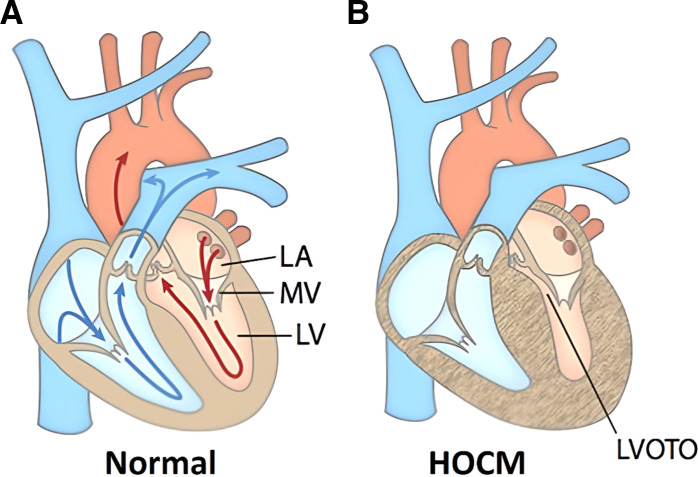
Difference in the anatomy between a normal and hypertrophic obstructive cardiomyopathy (HOCM) heart. *A*: normal heart where the arrows represent the direction of the blood flow. *B*: HOCM heart with an increased wall thickness, reduced left ventricular volume, and left ventricular outflow tract obstruction (LVOTO). LA, left atrium; LV, left ventricle; MV, mitral valve.

The aortic root (AR) is an important, complex organ that joins the left ventricle outflow tract (LVOT) with the ascending aorta and includes the aortic valve and the sinuses of Valsalva. The AR performs very sophisticated functions regulating the blood flow dynamics during the cardiac cycle. The AR has a complicated structure that is responsible for the development of vortices and maintaining normal functionality under different hemodynamic conditions; any pathological deformity in the structure impairs the functionality and propagation of blood flow, which can lead to life-threatening conditions. Thus, the AR plays a crucial role in ensuring the structural and functional synchrony to regulate the proper blood flow dynamics in several organs and in coronary flow (1–5). Such sophisticated functions depend on the symmetry and type of flow in the AR. In this study, we describe asymmetry of flow in the AR of healthy volunteers and compare it with the flow in patients with hypertrophic obstructive cardiomyopathy (HOCM), where we hypothesized maximal flow asymmetry in the AR flow because of the obstruction in the LVOT. Patients with HOCM are defined as patients with hypertrophic cardiomyopathy (HCM) with developed LVOT obstruction (LVOTO), which is a quantifiable feature of HOCM (6) and a prominent independent predictor of the severity of HCM (7–11), see Fig. 1 (12). It has been shown that the LVOTO in patients with HOCM perturbs the hemodynamics of the AR and the type of flow reaching the aorta (13–14) as well as the coronaries (11). The observations leading to this conclusion, however, have been largely qualitative.

Here, we report quantitative methods for measuring flow asymmetry in the flow of the AR. This could have clinical implications, which might be worthwhile to investigate for future clinical studies.

The main focus of this study is to quantify the asymmetry of flow in the AR in healthy volunteers compared with patients with HOCM. To the best of our knowledge, this is the first study to quantify the asymmetry of flow in the AR using two-dimensional (2-D) phase contrast magnetic resonance imaging (PC-MRI). These asymmetries could influence coronary flow as well as the perfusion of all other organs (1–5); the bulging shape of the aortic sinuses is responsible for the formation of vortices and blood recirculation during systole (15–16), which is crucial for feeding the coronary arteries and aids in valve closure (17). We hypothesize that changes in the vortices developments arising from the LVOTO in HOCM could eventually influence the perfusion of aortic blood flow to the coronaries as well as other organs. One of the several reasons behind the importance of studying AR flow is that the undesired backward flow from the aorta to the LV during systole could make the systolic forward flow inadequate and cause the heart to be prone to hypertrophy and elevated wall forces. This could put vital organs at risk, causing damages to the aortic wall and leading to life-threatening conditions.

In this study, we developed a method to characterize the pattern of flow in each sinus of Valsalva both at peak systolic flow and throughout the systolic phase. This enabled us to develop a quantitative assessment of asymmetry of flow in the AR, which could provide clinicians with quantitative data about their patients, both before and after relief of LVOTO (11).

## PATIENTS AND METHODS

### Data Acquisition

The data are derived retrospectively from 23 randomly chosen individuals: 13 healthy volunteers and 10 patients with HOCM before undergoing myectomy with a pressure difference exceeding 50 mmHg across the LVOT. Written informed consents were obtained from all individuals, see Table 1 for data demographics.

**Table 1.** Demographics data for healthy volunteers and patients with hypertrophic obstructive cardiomyopathy

Demographics	Healthy Volunteers (*n* = 13)	HOCM (*n* = 10)	*P* Value
Age, yr	28.53 ± 4.33	32.80 ± 9.86	0.14
Female, *n* (%)	3 (30%)	5 (38%)	0.75
BSA, m^2^	27.88 ± 4.40	28.78 ± 5.90	0.74
Weight, kg	79.00 ± 12.78	79.10 ± 18.03	0.96
Height, cm	168.81 ± 11.18	165.45 ± 4.81	0.67

Demographics data for 13 healthy volunteers and 10 patients with hypertrophic obstructive cardiomyopathy (HOCM). We used the Mann–Whitney *U* test to calculate the *P* values for the significance of the variables between cohorts. BSA, body surface area; *n*, number of females in the group.

Axial blood velocity was measured using 2-D PC-MRI. Four-dimensional (4-D) velocity measurements would have been preferable, but they were not available for all patients, unlike the 2-D PC-MRI that is commonly done as part of the clinical routine. MR images of the anatomy and PC-MR images of the blood velocity were acquired using a Magneton Aera 1.5 T scanner (Siemens, Germany), along with retrospective ECG triggering to capture the entire cardiac cycle. The repetition time/echo time was 3.6/1.8 ms, with a sense factor of 2, flip angle of 60°, section thickness of 8 mm, matrix of 160 × 256, field of view of 300 mm, pixel size of 1.6 × 1.6 mm, and 30 phases per cardiac cycle. The through-plane 2-D PC-MRI images were captured at the AR level in the middle of the sinuses of Valsalva.

### In-House Program for an Automated Assessment of the 2-D AR Flow

We developed a MATLAB (RRID:SCR_001622) program to study the AR flow across each sector in a less subjective and less time consuming approach to overcome some limitations that we found in the previously available software (18). We also present quantitative variables that were not previously available to quantify the skewness of the aortic jet in healthy volunteers and compare these measurements in patients with HOCM.

To investigate the different hemodynamics variables in the AR, the operator selects a rectangular region of interest (ROI) in the first 2-D magnitude cardiac magnetic resonance (CMR) image, corresponding to the peak of the R-wave in the ECG, which contains the AR lumen to be segmented. This region is applied to all the succeeding phase contrast images from which we calculate the 2-D axial velocity maps throughout the cardiac cycle. We also developed an objective method to divide the segmented AR lumen into the three sectors delineating the three sinuses of Valsalva, to study the flow in each sector.

**Figure 2.**
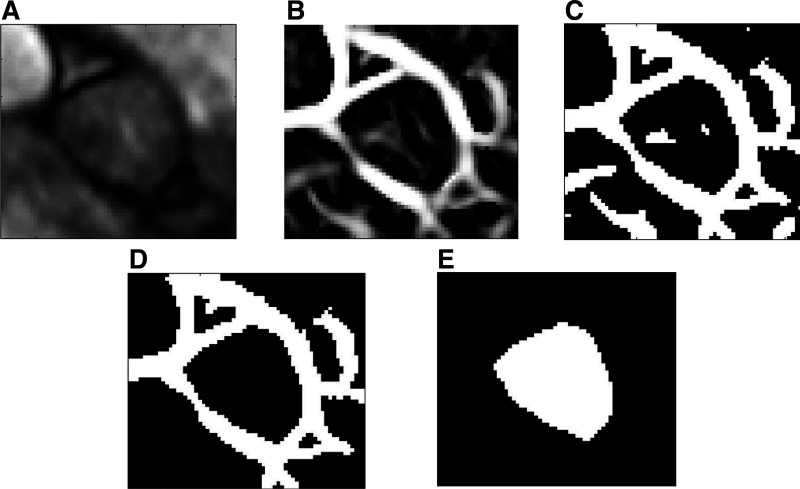
Segmentation of aortic root (AR) lumen. *A*: region of interest segmented from the two-dimensional (2-D) cardiac magnetic resonance magnitude image. *B*: the enhanced image of the AR lumen referred to as “V image,” which is used as a mask in the further analyses of the 2-D velocity image. *C*: binarization of the “V image” to remove vessels with low threshold. *D*: removal of connected objects that have few pixels; in our case, we used 200 pixels. *E*: the final segmented AR lumen.

As shown in Fig. 2, the AR lumen is primarily segmented using a previously described function (19) that enhances the vascular structure of the image by Hessian-based filters. In our study, we refer to the enhanced binarized vascular structure of the AR lumen as the “V image.” We first apply a threshold to the “V image” to remove vessels with low threshold, and we further improve this effect by removing all the remaining small connected objects with less than 200 pixels using “bwareaopen” as shown in Fig. 2*D*. We then use “bwconncomp” to study the connected components found in the binary image to mask out everything but the dark connected area found in the center of the image that represents the aortic lumen as shown in Fig. 2*E*.

The successful rate of the automatic segmentation that does not need further manual adjustment is 80%. If the final image is not well segmented and needs further optimization, then the user can select other segmentation options from the following.

1) Thresholding the cropped ROI: It allows the user to change the threshold of the cropped ROI and test its effect online on the segmented lumen from the cropped ROI, so that different thresholds of the original image can be tested. This option can be chosen if the predefined threshold of the “V image” results in a disconnected AR lumen.
2) Thresholding the “V image”: It is a slider to change the thresholds of the “V image.” This option can be chosen if the AR lumen is well connected and segmented but still needs slight optimization.
3) Manually segment the “V image”: It allows the manual segmentation of the AR lumen in the shown image, which can be applied in case the previous options do not give satisfactory segmentation.

The segmentation is done on each cardiac frame individually, and the time dimension was added by providing 30 frames every 25 ms. The total time needed to segment the 30 cardiac frames per subject depends on the resolution of the CMR images (around 2 min in high resolution instead of 20 min using the previous tool).

After the segmentation of the AR lumen over the cardiac cycle, the binary image of the lumen is used as a mask on the corresponding phase image to segment out the intensity values (δ) used in calculating the axial velocities in the AR lumen through the following (20–22):

$$U=-venc\left(\frac{\delta \zeta }{\gamma }+1\right)$$

In our MRI machine, the rescale intercept γ = −4096, rescale slope ζ = 2, and the encoding velocity of the acquisition venc is set by the operator and in our subjects was in the range of 180–350 cm/s.

**Figure 3.**
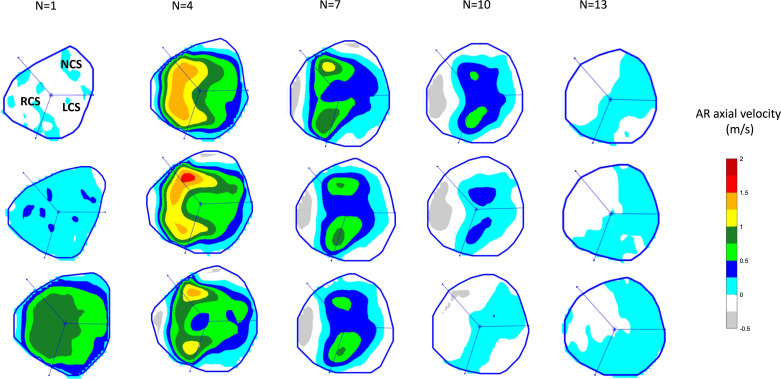
Example of dividing the aortic root (AR) lumen into the three sectors delineating the three sinuses of Valsalva (using our method that depends on the three edge points with the least distances from the centroid of the lumen) to show the axial velocity color maps of each sinus over the systolic phase, in a patient with hypertrophic obstructive cardiomyopathy (HOCM). The systolic phase ends after frame 10 in this patient with HOCM. LCS, left coronary sinus; N, cardiac frames; NCS, noncoronary sinus; RCS, right coronary sinus.

We present the calculated velocity values as color maps over the cardiac period. Figure 3 shows the measured velocity values in a patient with HOCM over the systolic phase; the velocity in the diastolic phase is almost zero.

### Identifying the Sinuses of Valsalva

We developed an automated method that divides the segmented AR lumen into the three sectors delineated by the three aortic sinuses of Valsalva to study the flow dynamics in the right coronary sinus (RCS), left coronary sinus (LCS), and noncoronary sinus (NCS). To do so, we calculate the distance and angle between the centroid of the AR lumen and each edge point; the angle being defined relative to the horizontal reference line defined by the MRI machine to get a polar plot for each frame. We smooth these plots with a Savitzky–Golay polynomial filter (second order with a sliding window of 15% of the length of the samples). From this, we determine the angles of the three edge points with the local minimum distances, since the sinuses are defined by the commissures (which are at the narrowest part of the sinus). In most subjects, there is some variation of the calculated sectors from the different time frames, indicating that there is some twisting and deformation of the AR during the cardiac cycle. However, the variation was small compared with the uncertainty in the identification of the commissures from the amplitude images by an experienced operator. We, therefore, defined fixed sectors as the median of the sectors during the cardiac cycle. The successful rate of the automated division of the AR lumen into the three sinuses of Valsalva is 100%. Figure 3 shows the three sectors superimposed on the color maps of the axial velocity during systole in a patient with HOCM.

### Investigating Different Hemodynamic Variables

To calculate the different flow parameters in the AR or in each sinus of Valsalva, we let M represent the set of all the pixels in the lumen, which is segmented into the sets L, R, and N (left, right, and noncoronary sinus) by the sectors. For each cardiac frame, we calculate the flow rate $\;Q_{Y}$ in the lumen and the three sectors by summing the flow rate in each pixel *j*:

$$Q_{Y}=\sum_{j\in Y\;}Q_{j\;}=\sum_{j\in Y}^{Y}U_{j}A_{j\;}$$

where *Y* is M, L, R, or N as appropriate. *U_j_* is the axial velocity; *A_j_* is the area of each pixel *j*, which is the same for each pixel in the lumen (2.56 mm^2^).

And we calculate the effective area of the aortic jet *A*_M_ using:

$$A_{M}=\;\frac{Q_{M}}{U_{M}}$$

where M represents the set of all the pixels in the lumen.

We also measure the fractional area of backward flow $\varphi_{Y}$ for the AR and for each sector:

$$\varphi_{Y}=\;\frac{N_{Q_{j}<0}}{N_{j}}$$

where *j* ∈ *Y*, $N_{Q_{j}}$< 0 represents the number of pixels that have negative flow values, and *N_j_* is the total number of pixels.

In addition, we define a flow-normalized asymmetry vector. Its magnitude is:

$$\alpha =|\frac{U_{CG}-C_{CG}}{R_{AR}}|$$

where, *U*_CG_ is the velocity center of gravity, *C*_CG_ is the area center of gravity (centroid), and *R*_AR_ is the mean radius of the AR lumen.

And its angle (the skewness angle) is:

θ = ∠($L_{CG}\;$ $L_{LN}$)

where *L*_CG_ is the line that connects *C*_CG_ with *U*_CG_ and *L*_LN_ is the line dividing the LCS and NCS.

#### Flow variables at peak systolic flow.

We used the flow rate to identify peak systolic flow within each subject and study the different flow variables in the AR at peak systole; we investigated $\hat{U}$_AR_, $\hat{Q}$_AR_, $\hat{A}$_AR_, $\hat{\varphi }$_AR_, and the variables that describe asymmetry of the AR flow, $\hat{\alpha }$ and $\hat{\theta }$. To further understand the statistical difference in the AR hemodynamics between cohorts at peak systolic flow, we also studied the different hemodynamics variables within the three sinuses of Valsalva: $\hat{U}$_L,N,R_, $\hat{Q}$_L,N,R_, and $\hat{\varphi }$_L,N,R_.

#### Flow variables averaged over systole.

To quantify the difference of the hemodynamic variables between cohorts throughout the systolic phase, we calculated the average over time during systole for each variable in each subject (accounting for the different duration of systole in different subjects), and we compared the average of these averages over the two cohorts for the different variables. We investigated the different hemodynamic variables in the AR throughout the systolic phase, such as $\bar{U}$_AR_, $\bar{Q}$_AR_, $\bar{A}$_AR_, $\bar{\varphi }$_AR_, $\bar{\alpha }$, and $\bar{\theta }$. We also studied $\bar{U}$_L,N,R_, $\bar{Q}$_L,N,R_, and $\bar{\varphi }$_L,N,R_ in the three sectors that correspond to the three sinuses of Valsalva (LCS, NCS, and RCS) throughout the systolic phase.

### Statistical Analysis

Data are expressed as means ± SD for the different parameters. For data comparison between patients with HOCM and healthy volunteers, we used the Mann–Whitney *U* test. A *P* < 0.05 is considered statistically significant, which indicates that the observations from patients with HOCM differ from those from healthy volunteers.

## RESULTS

Before proceeding with the 2-D PC-MRI for the AR flow analysis for all patients and healthy volunteers, we have initially studied the AR flow dynamics using the available 4-D flow measurements for two of the patients. We have found comparable results: a highly perturbed AR flow, with a skewed aortic jet rather than a more centralized aortic jet (a feature of the normal AR flow). This has been suggested by the negative velocities measured in the LCS—through calculating the spatial average velocity in each sinus of Valsalva at the AR level during peak systole—using the 4-D flow CMR images.

### Qualitative Assessment

**Figure 4.**
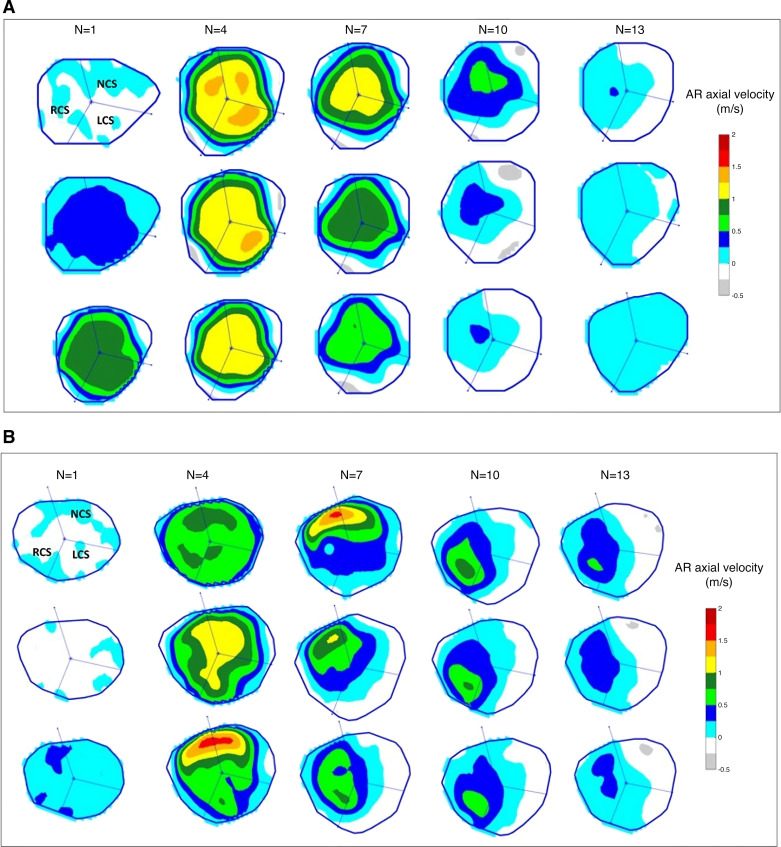
Axial velocity flow mapping of the aortic root (AR) and three sinuses of Valsalva over the first 15 cardiac frames including the systolic phase in a healthy volunteer (*A*) and in a patient with hypertrophic obstructive cardiomyopathy (HOCM; *B*). The systolic phase ends after *N* = 14 in this patient with HOCM and *N* = 10 in this healthy volunteer. LCS, left coronary sinus; N, frame number; NCS, noncoronary sinus; RCS, right coronary sinus.

We first studied the axial velocity flow mapping in the three sinuses of Valsalva in the form of color maps over the temporal frames constituting the systolic phase in our cohorts. Taking into account the difference in the length of the systolic phase among subjects, we determine the end of the systolic phase as the time when the total flow rate across the AR first reaches zero or below; in the figures, we show the first 15 cardiac frames to ensure that all the systolic frames are covered, and we present them in three rows for the sake of display to avoid taking too much space. The most dominant flows occur during systole when the aortic valve is open, and so we concentrate on the systolic phase. Figure 4 shows the axial velocity color maps of the AR divided into the LCS, NCS, and RCS sectors over the systolic phase in a healthy volunteer compared with a patient with HOCM. The degree of perturbation of the AR flow can be seen by comparing the flow color maps for a healthy volunteer (Fig. 4*A*) with the color maps for patients with HOCM (Figs. 3 and 4*B*).

### Quantitative Assessment

#### Flow variables at peak systolic flow.

##### Aortic root flow variables.

Our results at peak systole show that the peak axial velocity of the AR $\hat{U}$_AR_ is lower in healthy volunteers than in patients with HOCM, at *P* < 0.001, whereas the total flow rate $\hat{Q}$_AR_ and the effective area of the aortic jet $\hat{A}$_AR_ are larger in healthy volunteers at *P* < 0.01 and *P* < 0.001, respectively. We also found that the flow asymmetry index $\hat{\alpha }$ is lower in healthy volunteers at *P* < 0.03, see Table 2.

**Table 2.** Hemodynamics variables at peak systolic flow for the aortic root and each sinus of Valsalva

Peak Systolic Flow (Means ± SD)				
	Variables	Healthy Volunteers (*n* = 13)	HOCM (*n* = 10)	*P* Value
				
AR	$\hat{U}$_AR_, m/s	1.32 ± 0.14	1.66 ± 0.26	*<*0.001
	$\hat{Q}$_AR_, mL/s	4.48 ± 0.77	3.62 ± 0.75	*<*0.01
	$\hat{A}$_AR_, m^2^	3.48 ± 0.63	2.24 ± 0.63	*<*0.001
	$\hat{\varphi }$_AR_, %	6 ± 6	11 ± 10	0.18
	$\hat{\alpha }$, %	7 ± 6	16 ± 9	*<*0.03
	$\hat{\theta }$, degree	46 ± 110	97 ± 82	0.30
LCS	$\hat{U}$_L_, m/s	1.26 ± 0.16	1.21 ± 0.41	0.87
	$\hat{Q}$_L_, mL/s	1.48 ± 0.37	0.93 ± 0.37	<0.001
	$\hat{\varphi }$_L_, %	3 ± 7	14 ± 17	0.07
NCS	$\hat{U}$_N_, m/s	1.26 ± 0.12	1.44 ± 0.26	<0.04
	$\hat{Q}$_N_, mL/s	1.66 ± 0.57	1.22 ± 0.36	0.10
	$\hat{\varphi }$_N_, %	8 ± 8	12 ± 10	0.36
RCS	$\hat{U}$_R_, m/s	1.30 ± 0.14	1.14 ± 0.85	0.82
	$\hat{Q}$_R_, mL/s	1.34 ± 0.41	1.47 ± 0.54	0.33
	$\hat{\varphi }$_R_, %	5 ± 9	7 ± 10	0.47

Hemodynamics variables at peak systole (means ± SD) for the aortic root (AR) and each sinus of Valsalva for 13 healthy volunteers and 10 patients with hypertrophic obstructive cardiomyopathy (HOCM). We used the Mann–Whitney *U* test to calculate the *P* values for the significance of the hemodynamics variables between cohorts. $\hat{U}$, peak axial velocity; $\hat{Q}$, flow rate; $\;\hat{A}$_AR_, effective area of the aortic jet; $\hat{\varphi }$, fractional area of backward flow; $\hat{\alpha }$, flow asymmetry of AR jet; $\hat{\theta }$, skewness angle of AR jet; LCS, left coronary sinus; NCS, noncoronary sinus; RCS, right coronary sinus.

##### Sector flow variables.

As also shown in Table 2, the flow rate in the LCS $\hat{Q}$_L_ is significantly lower in patients with HOCM compared with healthy volunteers at peak systolic flow at *P* < 0.001. And the peak velocity in the NCS sector $\hat{U}$_N_ is larger in patients with HOCM compared with healthy volunteers at *P* < 0.04.

#### Flow variables averaged over systole.

##### Aortic root flow variables.

Our results averaged over systole show that the flow rate of the AR $\bar{Q}$_AR_ and the effective area of the aortic jet $\bar{A}$_AR_ are significantly higher in healthy volunteers compared with patients with HOCM at *P* < 0.001. We also found that the fractional area of backward flow measured in the AR $\bar{\varphi }$_AR_ and the flow asymmetry index $\bar{\alpha }$ are significantly reduced in healthy volunteers at *P* < 0.003 and *P* < 0.005, respectively, see Table 3.

**Table 3.** Hemodynamics variables averaged over systole for the aortic root and each sinus of Valsalva

Average over Systole (Means ± SD)				
	Variables	Healthy Volunteers (*n* = 13)	HOCM (*n* = 10)	*P* Value
AR	$\bar{U}$_AR_, m/s	0.84 ± 0.10	0.90 ± 0.16	0.33
	$\bar{Q}$_AR_, mL/s	2.50 ± 0.42	1.83 ± 0.24	*<*0.001
	$\bar{A}$_AR_, m^2^	3.03 ± 0.56	2.08 ± 0.48	*<*0.001
	$\bar{\varphi }$_AR_, %	12 ± 5	23 ± 5	*<*0.003
	$\bar{\alpha }$, %	9 ± 3	14 ± 5	*<*0.005
	$\bar{\theta }$, degree	81 ± 41	58 ± 52	0.25
LCS	$\bar{U}$_L_, m/s	0.80 ± 0.10	0.67 ± 0.18	0.05
	$\bar{Q}$_L_, mL/s	0.84 ± 0.24	0.49 ± 0.18	<0.001
	$\bar{\varphi }$_L_, %	8 ± 4	25 ± 12	<0.001
NCS	$\bar{U}$_N_, m/s	0.80 ± 0.11	0.71 ± 0.26	0.36
	$\bar{Q}$_N_, mL/s	0.90 ± 0.34	0.64 ± 0.23	<0.03
	$\bar{\varphi }$_N_, %	15 ± 6	23 ± 11	0.05
RCS	$\bar{U}$_R_, m/s	0.80 ± 0.10	0.61 ± 0.40	0.40
	$\bar{Q}$_R_, mL/s	0.76 ± 0.20	0.70 ± 0.16	0.47
	$\bar{\varphi }$_R_, %	15 ± 6	20 ± 10	0.16

The average over time during systole in each subject and comparing the average of these averages over cohorts ± SD for the different hemodynamic variables of the aortic root (AR) and across the three sinuses of Valsalva from 13 healthy volunteers and 10 patients with hypertrophic obstructive cardiomyopathy (HOCM). We used the Mann–Whitney *U* test to calculate the *P* values for the significance of the hemodynamic variables between cohorts. $\bar{U}$, peak axial velocity; $\bar{Q}$, Flow rate; $\bar{A}$_AR_, effective area of aortic jet; $\bar{\varphi }$, fractional area of backward flow; $\bar{\alpha }$, flow asymmetry of AR jet; $\bar{\theta }$, skewness angle of AR jet; LCS, left coronary sinus; NCS, noncoronary sinus; RCS, right coronary sinus.

##### Sector flow variables.

As also shown in Table 3, the flow rate averaged over systole in the LCS $\bar{Q}$_L_ and in the NCS $\bar{Q}$_N_ are larger in healthy volunteers when compared with patients with HOCM at *P* < 0.001 and *P* < 0.03, respectively. In addition, the fractional area of backward flow in the LCS averaged over systole $\bar{\varphi }$_L_ is significantly larger in patients with HOCM compared with healthy volunteers at *P* < 0.001.

## DISCUSSION

This study introduces several different hemodynamics variables to quantify the degree of asymmetry of flow in the AR that is normally assumed to be spatially regular and symmetric (23–24), although there are few, if any, studies demonstrating that this is the case. There are a number of factors that could cause asymmetry in flow in the AR (25): the left ventricle (LV) is anatomically asymmetric and its pattern of contraction is complex; the LVOT is anatomically complex; the aortic valve and the sinuses of Valsalva are superficially three-way symmetric, but the coronary arteries that divert ∼5% of the cardiac output originate in only two of the three sinuses (26–28); the ascending aorta is slightly curved and the aortic arch has a large and complex three-dimensional (3-D) curvature. In this study, we quantify the degree of asymmetry of the axial velocity in the AR at the level of the aortic valve in a cohort of healthy volunteers.

In contrast, flow in the AR is well known to be highly perturbed in patients with HOCM (13–14). The observations leading to this conclusion, however, have been largely qualitative based on visual imaging of the AR flow using ultrasound or MR imaging. In this study, we have explored how best to quantify flow at the level of the sinuses of Valsalva in the AR using axial velocity measurements from PC-MRI in a cohort of patients with severe HOCM awaiting myectomy. We have made rather detailed measurements throughout the cardiac cycle, but report mainly derived variables that can be measured relatively easily clinically.

The first variables we discuss are those that can be derived from images taken at a single time, specifically peak systolic flow, are shown in Table 2. The simplest of these variables are calculated from the whole lumen of the AR. We also show similar variables calculated for the three sectors delineated by the sinuses of Valsalva. These give a fuller picture of AR hemodynamics but involve more effort to enable the segmentation of the individual sinuses. We use a tilde to indicate variables measured at peak systolic flow.

We also looked at time-resolved variables to reveal information about the dynamical nature of AR hemodynamics. Apart from a few observations in the text derived from these dynamic measurements, we show only the time average over systole of these variables in Table 3. Again, we show first the averaged variables calculated over the whole of the lumen of the AR and then the averages for each of the three sinuses. We use a bar to indicate variables averaged over systole.

### Flow Variables at Peak Systolic Flow

For the variables measured using data from the entire lumen of the AR, the statistically most significant differences between healthy volunteers and patients with HOCM are the peak velocity $\hat{U}$_AR_, the flow rate $\hat{Q}$_AR_, and the effective area of the aortic jet *Â*_AR_. The increase of $\hat{U}$_AR_ while the volume flow rate $\hat{Q}$_AR_ is decreasing indicates that the jet entering the AR through the aortic valve is much narrower in HOCM than it is normally. The ratio of these two variables, $\frac{\hat{Q}}{\hat{U}}$, defined as *Â*_AR_ could be a convenient measure of the effective jet area, which is significantly reduced in patients with HOCM.

The flow asymmetry index at peak systolic flow $\hat{\alpha }$, which is relatively easily calculated, does differentiate between healthy volunteers and patients with HOCM with *P* < 0.03. The information gained from calculating the skewness angle, however, is minimal. The fractional area of backward flow at peak systolic flow is another parameter that is easy to calculate from the axial velocity measurements. It is approximately half as large in healthy volunteers compared with patients with HOCM, but because of the relatively large standard deviations in both cohorts, this variable is not statistically useful for differentiating HOCM.

The most prominent differences in the variables calculated in the three sectors delineated by the three sinuses is the fractional area of backward flow in the LCS $\hat{Q}$_L_ and the peak velocity in the NCS sector $\hat{U}$_N_. This indicates that the AR jet is directed on average into the NCS, something that is not clear statistical from the angle of the vector connecting the centroid of the lumen to the center of gravity of the axial velocity $\hat{\theta }$.

### Flow Variables Averaged over Systole

Measuring the axial velocity during systole provides much more data about the highly dynamical flow in the AR. We analyzed these data in some detail and found that the dynamic flow patterns in healthy volunteers are fairly regular, but in patients with HOCM, they are so irregular that is impossible to make statistically meaningful comparisons based on our small cohorts. However, some statistically meaningful results emerged when we averaged the measured flow variables over the duration of systole, as shown in Table 3.

The first of these statistically significant results, that the average flow rate in the AR $\bar{Q}$_AR_ is larger in healthy volunteers than in patients with HOCM, is expected because it is well known that the patients with HOCM have a reduced cardiac output. Interestingly, this expected difference between cohorts is higher for the average over systole, reaching 27%, when compared with difference of 19% calculated at peak systole.

Another averaged variable that shows a significant difference between healthy volunteers and patients with HOCM is the fractional area of backward flow $\bar{\varphi }$_AR_. This variable is a measure of the extent of recirculation in the AR due to the vortices generated by the starting jet during early systole. This systolic reversed flow has an important effect on coronary flow particularly during fast heart rates (1, 29). In addition, we have previously shown that there might be a correlation between AR flow and coronary in patients with HOCM (30). The fractional area of backward flow in HOCM is approximately double that in healthy volunteers, indicating that the aortic jet is narrower and the starting vortices are stronger in HOCM. Interestingly, the average over systole of this parameter is approximately double the value measured at the time of peak systolic flow in both cohorts. It is the reduction in the standard deviations for the averages over systole that is responsible for making this variable more significant.

The final variable that shows a meaningful difference between the healthy volunteers and the patients with HOCM is the average flow asymmetry index $\bar{\alpha }$. Again the averages of this variable over the two cohorts are similar to the averages of this parameter measured at the time of peak systolic flow, but the decrease in the standard deviations for the averaged variable decreases the *P* value by an order of magnitude. This makes us much more confident that the difference between the two cohorts is statistically meaningful. This nondimensional variable indicates how far “off-center” the aortic jet is in the AR. Our results show that the aortic jet is not perfectly symmetrical in healthy volunteers, which is not very surprising given the rather complex anatomy of the LVOT and the AR. In HOCM, this index is nearly double that in healthy volunteers, and we suggest that it would be a useful thing to measure in the characterization of the severity of HOCM.

The averages over systole of the variables measured in the individual sectors, particularly in the LCS, follows the pattern for the variables measured in the whole AR lumen; the average flow rate in the LCS $\bar{Q}$_L_ and the fractional area of backward flow in the LCS $\bar{\varphi }$_L_ are highly significantly different between HOCM and healthy volunteers, indicating that the difference between cohorts in the total flow rate $\bar{Q}$_AR_ and the fractional area of backward flow in the whole lumen of the AR $\;\bar{\varphi }$_AR_ is mainly due to the differences in the LCS.

### Limitations

One of the limitations in our study is that we have a small number of subjects, which means that all our clinical results should be taken as preliminary. Our measurements were done using 2-D PC-MRI not 4-D velocity measurements. Despite the comprehensive measurements given by the 4-D flow for the three-directional velocity of blood flow, there are some limitations in the 4-D flow that need to be addressed: it lacks standardized analytical methods and quantitative measurements for a reproducible and comparable results to ease its translation to clinical practice. Besides, the 4-D analysis is complex, increases the scan time, and has less temporal resolution. For these reasons and because the 2-D PC-MRI is commonly done as part of the clinical routine, hence available for all our subjects, we decided to use the through-plane 2-D PC-MRI to study the spatial and temporal distribution of the blood flow for the axial component of the velocity in 10 patients with HOCM and 13 healthy volunteers.

Also, the segmentation of the AR lumen and its division into the three sinuses of Valsalva could be affected by the low MRI resolution. If it is possible, it would be better to use high-resolution MRI acquisition to capture the geometry of the AR lumen accurately. Our findings and interpretations would have been stronger and invaluable to surgeons if we could study AR hemodynamics in post-myectomy patients as well.

### Conclusions

We have studied the pattern of AR hemodynamics in healthy volunteers compared with patients with HOCM and found clinically convenient ways to quantify asymmetry of flow in the AR. We present an algorithm for the separation of the AR lumen into sectors corresponding to the sinuses of Valsalva, which involves a small amount of additional work, whose benefits are apparent when the variables are averaged over systole. The percentage of backward flow in the sinuses of Valsalva calculated over systole is a potential indicator of perturbed AR hemodynamics and the distribution of vortical flow, and it could be used as a measure of flow asymmetry. These measurements from the individual sectors could be very informative when assessing the effect of myectomy on the flow in the AR. They also suggest the possibility of an interaction between AR and coronaries, which should be studied further. It would be very informative if future studies involve the measurement of the LV performance, since the deranged flow in the AR could affect it directly or through an effect on coronary flow and thus LV perfusion.

## DATA AVAILABILITY

Data will be made available upon reasonable request.

## DISCLOSURES

No conflicts of interest, financial or otherwise, are declared by the authors.

## AUTHOR CONTRIBUTIONS

N.F., M.H.Y., and K.H.P. conceived and designed research; N.F. and M.H. performed experiments; N.F. analyzed data; N.F., M.H.Y., and K.H.P. interpreted results of experiments; N.F. and M.H. prepared figures; N.F. drafted manuscript; N.F., M.H.Y., and K.H.P. edited and revised manuscript; N.F., M.H., M.H.Y., and K.H.P. approved final version of manuscript.
